# Genetics and genomic medicine around the world

**DOI:** 10.1002/mgg3.59

**Published:** 2014-01-10

**Authors:** Suzanne Hart, Maximilian Muenke

**Figure:**
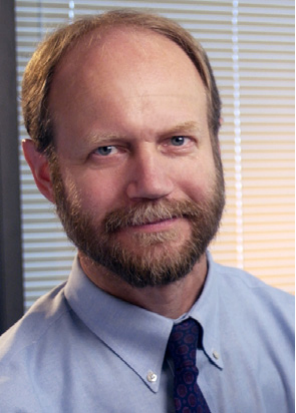


**Figure:**
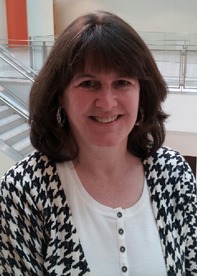


As we unveil our first issue of 2014, we would like to announce the launch of a new feature of *Molecular Genetics and Genomic Medicine*: “Genetics and Genomic Medicine around the World.” These articles will focus on various aspects of genetics and genomic medicine internationally. Trends in different countries will be described by a senior colleague from that country.

In 1988, the *Journal of Medical Genetics* launched a similar series entitled “Medical Genetics around the World” (Harper 1988). The purpose of the series was to highlight the delivery of genetic services in various countries. As noted by Dr. Harper, “the state of medical genetics is critically dependent on the surroundings in which it is placed, reflecting not just the economic and medical development of a country, but its social attitudes and the organization of medical services.” According to Dr. Harper, the “joy of being an editor [for the *Journal of Medical Genetics*] was the freedom to develop and write about anything that was interesting to [him], and with the idea to make things as international as possible,” he decided to develop this series (personal communication, December 2013). Over a period of 6 years, five articles overviewing medical genetics in China (Hui-Yuan 1988), Hong Kong (Chau et al. 1990), Hungary (Czeizel 1988), Israel (Goodman et al. 1989), and South Africa (Jenkins 1990) were published. Each article included a brief history of medical genetics in that country. Additional content varied from article to article, but included information on laboratory testing, prenatal diagnosis, genetic counseling, and training in medical genetics. The series also included articles on recessive disorders in Arabs (Teebi 1994), gene mapping in Finland (de la Chapelle 1993), and molecular diagnosis in Russia and the former USSR (Baranov 1993).

*Genetics in Medicine* initiated a series in 2001 entitled “Human Genetics around the World” with the intention to inform medical genetics practice: “While operating in environments that are politically and culturally different, we all can learn from each other's experiences” (Francke 2001). This series focused on genetics societies in Great Britain (Haites 2002), Greece (Tsenghi and Tzeli-Kitsiou 2001), and Portugal (Saraiva et al. 2001), as well as an article focusing on genetic discrimination in Australia (Otlowski et al. 2002).

Given that two other journals have attempted a similar series, why establish this feature in *Molecular Genetics and Genomic Medicine* now? First, more than ever, medical genetics is a global practice. Samples are sent to laboratories around the world for diagnostic testing. The Genetic Testing Registry (http://www.ncbi.nlm.nih.gov/gtr/) currently includes laboratories from 39 countries. Understanding clinical practice in one country may lead to altered practice in another country. This may be especially important in working with immigrant populations and understanding their views on genetics and genomics (Buseh et al. 2013). Second, genetics continues to be a rapidly changing field in which we are now learning to interpret much larger amounts of data from next-generation sequencing. Besides the bioinformatics challenge of big data, the ethical debate on which next-generation incidental results to return to patients has left many leaders in our field on opposite sides of the fence (Burke et al. 2013). As we are coming to grips with secondary findings in developed countries, there is uncharted territory in many areas of the globe such as sub-Saharan Africa, which may not have the medical infrastructure to evaluate incidental findings. We believe that this series will be an invaluable sounding board for learning how different countries and cultures view and adopt genetic and genomic testing.

The internet and social media play a major role in the dissemination of genetic information and connecting the worldwide genetics community. Twenty years ago, who would have predicted that there would be apps to help individuals determine if they are closely related to an individual with whom they are contemplating starting a relationship (Wright 2013). The internet also facilitates a way to advertise and promote genetic testing, especially direct to consumer genetic testing. Learning about practices and resources in other countries can help all of us be better geneticists and benefit our patients. This series will also include information on genomics in each of the highlighted countries. We envision that this series will present a concise overview of the current state of medical genetics and genomic medicine practice, including a brief history, highlighting accomplishments, training of geneticists, and the future of the field.

Given the diverse cultural practices and perceptions of genetic testing, many ethical issues need to be taken into consideration when performing genetic testing in some countries. One section will be devoted to Ethical, Legal, and Social Implication (ELSI) issues of particular importance in that county, focusing on disorders found with high prevalence in the population, attitudes toward genetic counseling and testing in the country, etc.

We hope that you will enjoy learning about the history and practice of genetics and genomic medicine in other countries. Please contact us if you wish to prepare the article for your country!
